# *Lavandula dentata* L.: Phytochemical Analysis, Antioxidant, Antifungal and Insecticidal Activities of Its Essential Oil

**DOI:** 10.3390/plants11030311

**Published:** 2022-01-25

**Authors:** Youness El Abdali, Abdelkrim Agour, Aimad Allali, Mohammed Bourhia, Abdelfattah El Moussaoui, Noureddine Eloutassi, Ahmad Mohammed Salamatullah, Abdulhakeem Alzahrani, Lahcen Ouahmane, Mourad A. M. Aboul-Soud, John P. Giesy, Abdelhak Bouia

**Affiliations:** 1 Laboratory of Biotechnology, Environment, Agri-Food and Health, Faculty of Sciences Dhar El Mahraz, Sidi Mohammed Ben Abdellah University, Fez 30050, Morocco; abdelfattah.elmoussaoui@usmba.ac.ma (A.E.M.); bouia.abdelhak@gmail.com (A.B.); 2 Laboratory of Natural Substances, Pharmacology, Environment, Modeling, Health & Quality of Life, Faculty of Sciences Dhar El Mahraz, Sidi Mohamed Ben Abdellah University, Fez 30050, Morocco; abdelkrim.agour@usmba.ac.ma; 3 Laboratory of Plant, Animal and Agro-Industry Productions, Faculty of Sciences, University of Ibn Tofail, Kenitra 14000, Morocco; aimad.allali@uit.ac.ma; 4 Laboratory of Microbial Biotechnologies, AgroSciences and Environment, Labeled Research Unit-CNRST N° 4, Faculty of Sciences Semlalia, Cadi Ayyad University, Marrakesh 40000, Morocco; bourhiamohammed@gmail.com (M.B.); l.ouahmane@gmail.com (L.O.); 5 Laboratory of Pedagogy and Technological Innovation, Regional Centre of Education and Formation Professions, Fez 30050, Morocco; noureddine.eloutassi22@gmail.com; 6 Department of Food Science & Nutrition, College of Food and Agricultural Sciences, King Saud University, 11 P.O. Box 2460, Riyadh 11451, Saudi Arabia; asalamh@ksu.edu.sa (A.M.S.); aabdulhakeem@ksu.edu.sa (A.A.); 7 Department of Clinical Laboratory Sciences, College of Applied Medical Sciences, King Saud University, P.O. Box 10219, Riyadh 11433, Saudi Arabia; 8 Toxicology Centre, University of Saskatchewan, 44 Campus Drive, Saskatoon, SK S7N 5B3, Canada; jgiesy@aol.com; 9 Department of Veterinary Biomedical Sciences, University of Saskatchewan, Saskatoon, SK S7N 5B4, Canada; 10 Department of Integrative Biology, Center for Integrative Toxicology, Michigan State University, East Lansing, MI 48824, USA; 11 Department of Environmental Science, Baylor University, One Bear Place #97266, Waco, TX 76798-7266, USA

**Keywords:** sustainable, environmentally friendly, antiradical, biofungicide, bioinsecticide, pharmaceutical, insect repellent

## Abstract

Antioxidant, antifungal and insecticidal activities of essential oil (EO) extracted from the Moroccan lavender (*Lavandula dentata*) were investigated and their chemical constituents determined. Gas chromatography with flame ionization detection (GC-FID) and gas chromatography-mass spectrometry analyses (GC-MS) were used to examine the phytochemical composition of EO. Antioxidant potential was examined in vitro by use of three tests: DPPH inhibition, reducing power (FRAP) and total antioxidant capacity (TAC). Antifungal activity was assessed by calculating inhibition of growth of *Alternaria alternata*, *Botrytis cinerea* and *Fusarium oxysporum*. Repellent potential and toxicity of EO by contact and inhalation were performed against *Callosobruchus maculatus*. Sixteen constituents were detected in the EO of *Lavandula dentata*. The major component was linalool (45.06%) followed by camphor (15.62%) and borneol (8.28%). EO exhibited a significant antioxidant activity, as measured by DPPH and FRAP assays, with IC_50_ and EC_50_ values of 12.95 ± 1.300 mg/mL and 11.88 ± 0.23 mg/mL, respectively. EO of lavender exhibited total antioxidant capacity of 81.28 ± 2.28 mg AAE/g EO. EO of lavender showed an inhibitory effect on mycelial growth against tested fungi and was 100% in the case of *B. cinerea*. EO caused total mortality of adult *C. maculatus* from 5 µL/L air with LC_50_ value of 4.01 µL/L air. Significant reduction in numbers of eggs laid (99.2%) and emergence (100%) was observed in a dose-dependent manner up to maxima of 100% and 99.2%, respectively. EO of lavender also showed a moderate potency to repel insects with a mean of 34.44%. EO of Moroccan *Lavandula dentata* has potential to be an effective natural agent against free radical damage and could be an environmentally friendly alternative bio-fungicide and bio-insecticide.

## 1. Introduction

Filamentous fungi are the most common type of pathogenic microorganisms that reduce agricultural productivity and quality and cause major economic losses of crops [1]. Mycotoxigenic fungi are known for their ability to colonize a wide range of cereal grains, vegetables, spices and fruits. This type of mold produces mycotoxins, which are associated with various effects on health of humans, including genotoxicity, carcinogenicity and immunosuppression [2]. Currently, adoption of resistant cultivars and phytosanitary treatments are the main stays of prevention strategies to control infection by fungi [3]. To minimize accumulation of residues in soil and novel fungal races with resistance to chemical treatments, new environmentally friendly, crop protection approaches are required [4]. In this context, essential oils (EOs) offer potential alternatives to synthetic fungicides to improve protection measures against phytopathogenic fungi.

Due to its richness in dietary protein, minerals and vitamins, globally, chickpea (*Cicer arietinum*) is one of the most nutritious seed legumes for human consumption [5]. Losses of yields of leguminous crops during storage due to various types of insects, especially bruchids, is a serious problem for traders and farmers [6]. Chickpea weevil (*Callosobruchus maculatus*), is one of the most damaging pests of chickpea. This insect is able to lay eggs both in cultivated fields and storage areas. Its larvae, which feed internally on seeds, are difficult to control with chemical insecticides [5]. Synthetic insecticides are routinely used to control pests in agricultural crops. However, their indiscriminate and excessive usage has led to harmful consequences for human health, environmental pollution, residues of pesticides on fruits, vegetables and seeds, insect pests and weeds resistant to effects of crop protection chemicals. Currently, due to their specificity for agricultural pests, and biodegradability, biopesticides developed from EOs might be a viable option for protecting crops while reducing negative effects of synthetic pesticides [7].

Free radicals are chemical species with a single electron on their peripheral layer resulting from cellular oxidation in mammals. Free radicals can cause cytotoxic consequences and tissue lesions and also damage DNA. In order to defend itself, the human body needs antioxidant agents that neutralize these free radicals. Plants, through their secondary metabolites, provide powerful antioxidant agents to control and mitigate the harms of free radicals [8].

Recently, EOs produced from aromatic plants have attracted interest because of their many biological actions [9]. An EO is a concentrated hydrophobic liquid containing volatile, easily evaporated at normal temperatures, chemical compounds isolated from plants. Essential oils are also known as volatile oils, ethereal oils, aetheroleum, or simply as the oil of the plant, from which they were extracted. EOs of various species of *Lavandula dentata* (Lamiaceae) have a broad range of biological effects including sedative, antibacterial, antifungal, antidepressant, antioxidant and anti-inflammatory properties [10]. Due to its major terpenic components, such as linalool, camphor, eucalyptol, and linalyl acetate, these essences have also demonstrated considerable success for control of insects [7]. However, there is a lack of information and research on other bioactivities of EOs from the Moroccan variety of *L. dentata*, especially for insect pests in agriculture and some phytopathogenic fungi. Therefore, the purpose of this study was to characterize and assess antioxidant capacity and antifungal activity of EOs isolated from the aerial parts of *L. dentata*, against some phytopathogenic fungi implicated in contamination of leguminous products. The study also investigated insecticidal activity of EOs of this emblematic Moroccan plant pharmacopoeia against the chickpea weevil.

## 2. Results and Discussion

### 2.1. Yield of Essential Oil

Hydro-distillation of aerial parts of *L. dentata* gave a pale yellow-green solution of EO with a characteristic fresh odor and yielded 3.46%. The yield obtained is higher than that of *L. dentata* studied in Tunisia (1.76%) [2], and also Moroccan lavender (2.9%) [11].

### 2.2. Physical Parameters of the Essential Oil

Physical properties of *L. dentata* EO were used as parameters of quality according to recommendations set by the European Pharmacopoeia [12]. All parameters investigated complied with AFNOR standards (Table 1). This reflects the efficiency of the hydro-distillation method used.

### 2.3. Phytochemical Composition of Essential Oil

**Figure 1:**
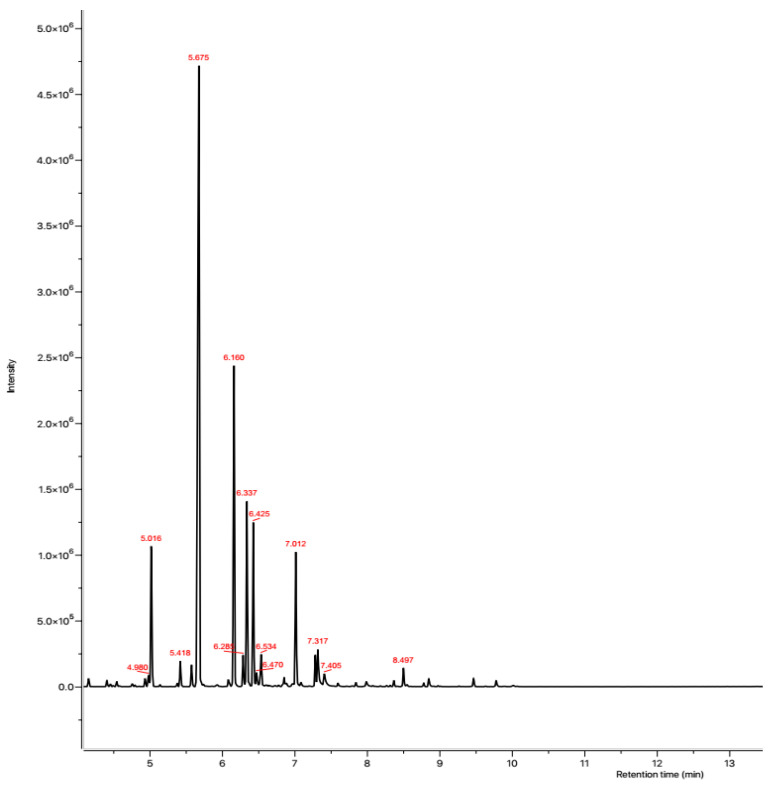
GC-MS chromatographic profile of *L. dentata* EO.

**Figure 2:**
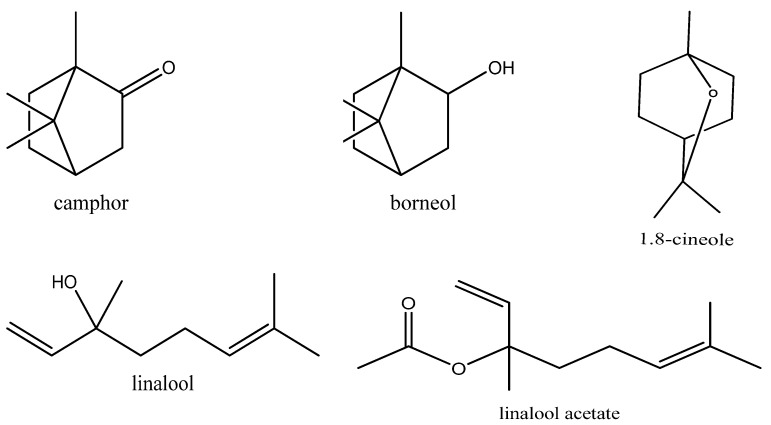
Molecular structure of phytochemical compounds of some molecules in *L. dentata* EO.

Using GC-FID and GC–MS techniques, the chemical composition of EO extracted from *L. dentata* was identified and quantified (Figure 1 and Figure 2). Constituents found are summarized in order of times of elution from the HP-5MS column (Table 2). Sixteen compounds were identified in *L. dentata* EO representing 99.61% of the total essence. The major constituent was linalool (45.06%) followed by camphor (15.62%), and borneol (8.28%), all of which belong to the class of oxygenated monoterpenes that constitute the major class (91.18%) of the studied EO. Sesquiterpene hydrocarbons were represented by only β-farnesene (0.86%) and other compounds comprised 7.57% of the EO of lavender. Other chemotypes of Moroccan lavender have been previously investigated. The 1,8 cineol (41.28%) was the major compound of *L. dentata* from eastern Morocco [13], while lavender collected from the Moroccan middle Atlas was rich in Camphor (49.75%) [14]. In Tunisia, EO extracted from leaves of *L. dentata* were rich in camphor (26.52%) and 1,8 cineole (22.90%) [15]. The 1,8 cineole was also observed to be a major constituent of lavender cultivated in Brazil (63.25%) [16], and in Italy (69.08%) [17]. These differences can be attributed to seasonal conditions, circadian rhythms, and environmental influences [16]. Some compounds in characterized EO including linalool, camphor, borneol, thymol and carvacrol are potentially bioactive with multiple pharmacological activities [18]. 

### 2.4. In Vitro Antioxidant Activity of Essential Oil

Antioxidant properties of *L. dentata* EO were assessed using DPPH scavenging, FRAP, and phosphomolybdenum tests (Table 3). When the DPPH test was conducted to evaluate the hydrogen-donating or radical-scavenging potential of the EO in the presence of 2,2-diphenyl-1-picrylhydrazyl (DPPH) stable radical as a reagent, the concentration of EO that caused a 50% inhibition (IC_50_) of the free radical was 12,950 ± 1300 mg EO/mL. The IC_50_ of the studied oil was significantly (*p* < 0.001) lesser than the value of the reference antioxidant (BHT), which had an IC_50_ value of 0.134 ± 0.028 mg/mL.

Essential oils are mixtures of several volatile and semi-volatile compounds with various polarity and functional groups, which results in a variety of chemical behaviors. Depending on the test utilized, these mixtures might give different results. It was, therefore, necessary to use a multi-assay approach to assess antioxidant ability of EO studied. In this regard, Results of the TAC test showed that the total antioxidant capacity of EO and reference antioxidants (BHT and quercetin) expressed as ascorbic acid equivalents (mg AAE/g EO) were 81.280 ± 2.278 mg/mL, 47.540 ± 1.200 mg/mL and 28.390 ± 1.248 mg/mL respectively (Table 3).

In the present study, the antioxidant potential was also assessed utilizing the FRAP assay (Table 3). Lavender EO were able to reduce Fe^3+^ to Fe^2+^ with an EC_50_ value of 11.880 ± 0.225 mg/mL, but this reducing capacity was found significantly (*p* < 0.001) less potent than those of the synthetic antioxidants BHT and quercetin with an EC_50_ values of 0.362 ± 0.010 mg/mL and 0.032 ± 0.003 mg/mL respectively.

Findings of the study, results of which are reported here, were compared with those published in previously, all of which exhibited lesser antioxidant potencies for *L. dentata* EOs based on various assays [2,16,19]. Several studies conducted on other species belonging to the genus *Lavandula* reported a significant antioxidant potential in the case of *L. pedunculata*, *L. stoechas* and *L. officinalis* [20,21]. Moderate antioxidant potential of the EO extracted from *L. dentata* is probably due to the complex mixture of compounds it contains and to the large proportion of oxygenated compounds (91.18%). Furthermore, unsaturated compounds, especially those with more than one hydroxyl group are more involved in antioxidant processes of lavender, in particular the ability to reduce the free radical DPPH [16]. Several studies conducted on antioxidant potencies of several compounds suggested that linalool and 1,8 cineole, which are major constituents of the lavender EOs, exhibited a moderate antioxidant potential compared to carvacrol, which is a minor compound in EOs of lavender [22]. Similar work has reported that linalool performs through several assays a stronger antioxidant potential compared to 1.8 cineole, camphor, borneol and terpinen-4-ol. While thymol and carvacrol were the strongest in terms of antioxidant activity [18]. Consequently, this diversity of chemicals from the studied EO, in addition to their potential action mode and interactions, make it difficult to attribute antioxidant effect to one or a few active compounds. Antioxidants can exhibit a wide range of biological effects, including anti-atherogenic, anti-allergic, antimicrobial, antioxidant and anti-inflammatory and their quantification and identification can be considered an important step to discover biological and chemical properties of this natural compounds [23].

### 2.5. Antifungal Activity of Essential Oil

**Figure 3:**
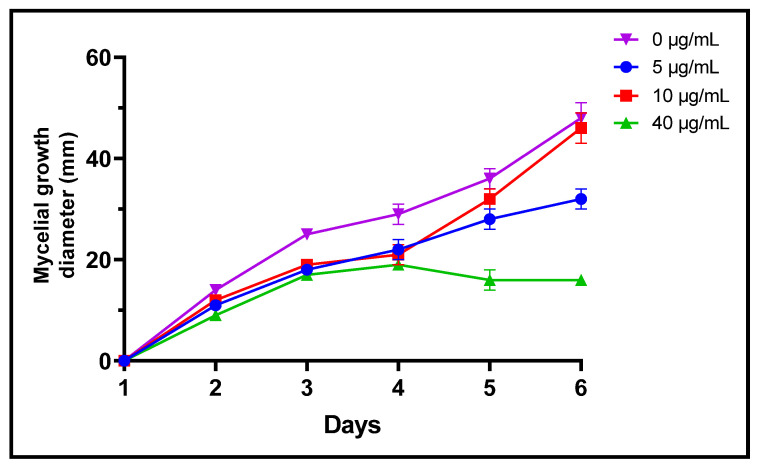
Mycelial growth kinetic of *A. alternata* treated by three concentrations of *L. dentate* EO.

**Figure 4:**
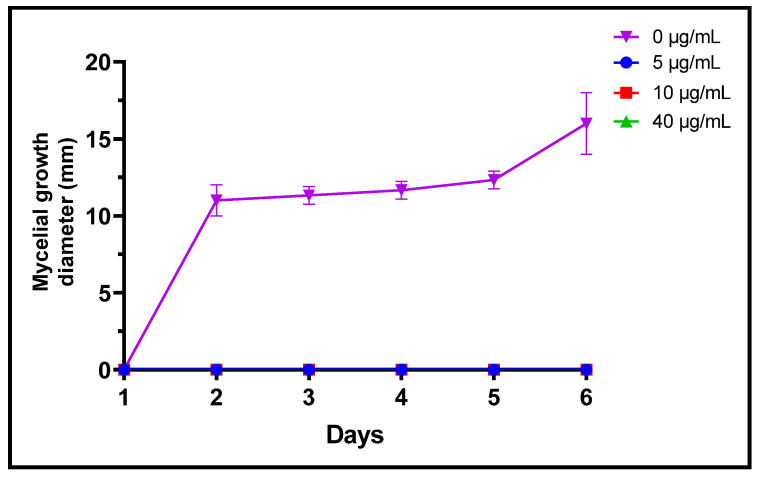
Mycelial growth kinetic of *B. cinerea* treated by three concentrations of *L. dentata* EO.

**Figure 5:**
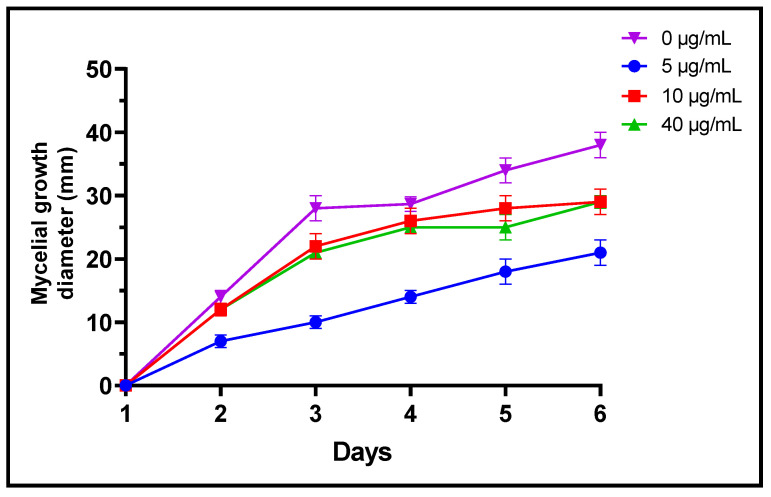
Mycelial growth kinetic of *F. oxysporum* treated by three concentrations of *L. dentata* EO.

Fungi are frequently implicated in the contamination of fruit and legume crops during harvest or storage, which is the case for the genera *Fusarium*, *Alternaria* and *Botrytis* [24]. These mycotoxin-producing filamentous molds are pathogenic and constitute a real danger to human health [2]. In the present study, three different concentrations of *L. dentata* EO were tested to evaluate, in vitro, their antifungal effects on growth of *B. cinerea*, *A. alternata* and *F. oxysporum* on Czapek-Dox agar medium (Figure 3, Figure 4 and Figure 5). Lavender EO exerted various antifungal actions on growth of the studied fungi and inhibited its mycelial growth in a dose dependent manner. During the entire incubation time, *L. dentata* oil totally inhibited *B. cinerea* mycelial growth at all doses tested. Oil concentrations of 5 and 10 μg/mL were ineffective against *A. alternata* mycelial growth, while the concentration of 40 μg/mL showed partial antifungal activity. Against *F. oxysporum*, all three concentrations, especially 5 μg/mL, exhibited an inhibitory effect compared to the control, by retarding the mycelial growth kinetics of the fungus, which started on the second day. 

**Figure 6:**
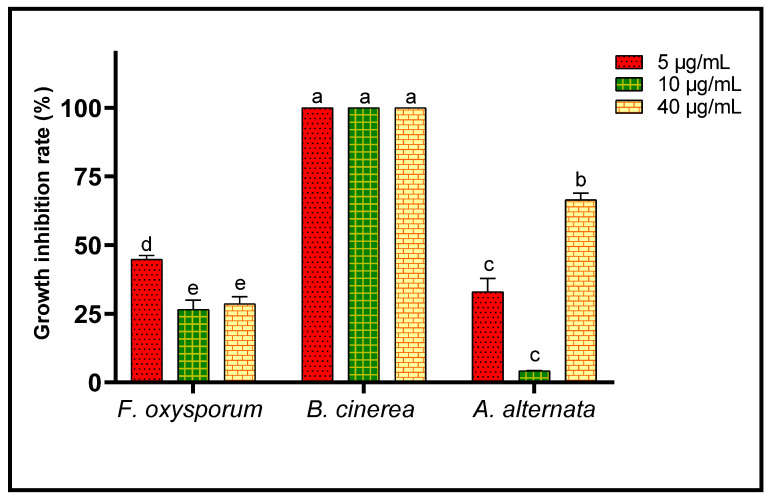
Growth inhibition rate of *A. alternata*, *B. cinerea* and *F. oxysporum* after the 6th day of treatment by three concentrations of *L. dentata* EO. For every fungal strain values with different letters are significantly different (*p* < 0.05).

Inhibition of growth of the three fungal strains studied was calculated after the 6th day of incubation for all concentrations of EO studied (Figure 6). *B. cinerea* seems to be the most sensitive to lavender EO, since growth of that fungus was completely (100%) inhibited. *F. oxysporum* and *A. alternata* were less sensitive to inhibition by EO of *L. dentata*, but growths of these fungi were partially inhibited. Thus, after 6 days of incubation, 40 μg/mL of the EO inhibited *A. alternata* growth up to 66.48 ± 2.41%. The same oil inhibited growth of mycelium of *F. oxysporum* by 44.81 ± 1.36% at a concentration of 5 μg/mL. The IC_50_ value corresponding to the EO concentration responsible for 50% inhibition of mycelial growth, after the 6th day of incubation, was also calculated for *F. oxysporum* (23.95 ± 2.83 μg/mL), whereas it was incalculable in the case of *B. cinerea* and *A. alternate*.

Results of this study are better than those in which exposure to 1 mg *L. dentata* EO/mL inhibited growth of mycelia of *A. alternata* and *S. cucurbitacearum* by 54% and 73.92% respectively [1] with higher IC_50_ values of 893 μg/mL and 389 μg/mL, respectively. It has been reported also that a concentration of 0.1% of *L. dentata* EO inhibited mycelium growth of *A. carbonarius* by 63.7% [2]. Results of other studies have shown that a concentration of 60 μL/disc of EO extracted from *L. stoechas* inhibited mycelium extensions of *F. oxysporum* and *R. solani* by 71.3–76.6% and 100%, respectively [25]. Antifungal effects of Eos are related to their chemical compositions. Potential individual or synergetic effects between the major and minor compounds can occur. Linalool, borneol, 1,8-cineole and camphor, which are major constituents of *L. dentata* EO, might be responsible for the observed fungicidal action. Results of other studies have indicated that linalool followed by 1,8-cineole have a strong toxic effect against growth of mycelia of *A. pullulans*, *D. hansenii* and four fungi of the genus *Penicillium* [22]. Furthermore, growth of mycelia and germination of spores of *Botrytis cinerea* in vitro were strongly reduced by application of carvacrol and thymol, which are minor constituents of the lavender EO studied here [26]. Consequently, this diversity of chemicals present in the studied EO, in addition to their various antifungal potency, make it difficult to attribute the antifungal action to one or more active components. Different mechanisms have been reported to explain the toxicity of EOs against fungi. Indeed, the lavender antifungal effect could be related to EO terpenes and phenolic chemicals, which are known to damage cell membranes, causing leakage of cellular materials, and responsible for the inhibition of electron transport and ATPase in the mitochondria and ultimately the microorganism death [27]. In closer works, it was stated that some EOs reduced significantly the production of the phospholipase enzyme produced by *C. albicans* strains decreasing strongly its virulence [28].

### 2.6. Insecticidal Activity of Essential Oil

#### 2.6.1. Toxicity of Essential Oil against *C. maculatus*

**Figure 7:**
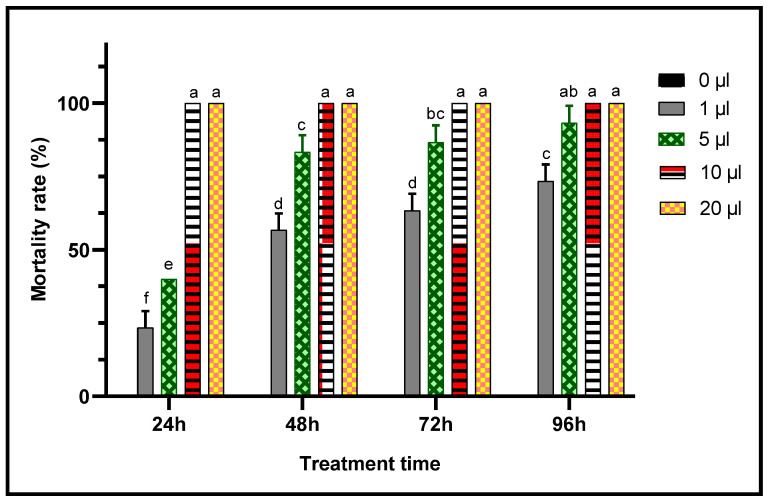
Mortality (means ± SD) of *C. maculatus* adults exposed to a contact toxicity test of different concentrations of *L. dentata* EO. Values with different letters are significantly different (*p* < 0.05).

**Figure 8:**
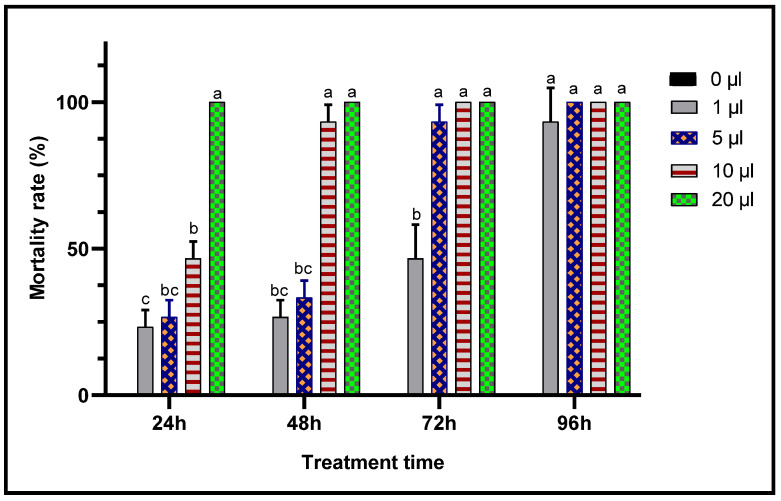
Mortality (means ± SD) of *C. maculatus* adults exposed to an inhalation toxicity test of different concentrations of *L. dentata* EO. Values with different letters are significantly different (*p* < 0.05).

When toxic potencies of *L. dentata* EO against *C. maculatus* adults was studied through two inhalation and contact tests to evaluate their insecticidal activity against this chickpea pest, lavender EO exhibited a very significant insecticidal effects in both contact and inhalation tests (Figure 7 and Figure 8). In fact, mortality of adult *C. maculatus* increased with greater doses of EO and durations of exposure. The least concentration (1 μL/L) of EO of *L. dentata* caused lethality 23.33 ± 5.77% of adult *C. maculatus*, in both tests studied. Mortality increased considerably (*p* < 0.05) with the duration of exposure up to 96 h. At greater concentrations, total mortality (100%) was observed from the first 24 h in chickpea bruchid adults treated with 10 μL/L and 20 μL/L of the EO in contact and inhalation tests respectively. No mortality (0%) was observed in the control jar.

Concentrations of the lavender EO that cause 50% (LC_50_) and 95% (LC_95_) mortality in *C. maculatus* adults during 24 h were also calculated (Table 4). The biocide effect was more important in the case of the contact test, for which the LC_50_ (LC_50_ = 4.01 μL/L air) was less than during the inhalation test (LC_50_ = 5.90 μL/L air).

#### 2.6.2. Effects of Essential Oil on Fecundity and Emergence of *C. maculatus*

**Figure 9:**
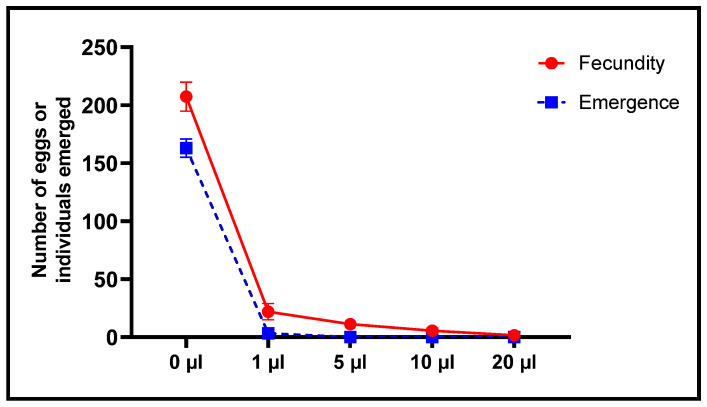
Fecundity of females (mean values of eggs laid ± SD) and Emergence (means ± SD) of *C. maculatus* adults after a direct contact toxicity test with different concentrations of *L. dentata* EO.

**Figure 10:**
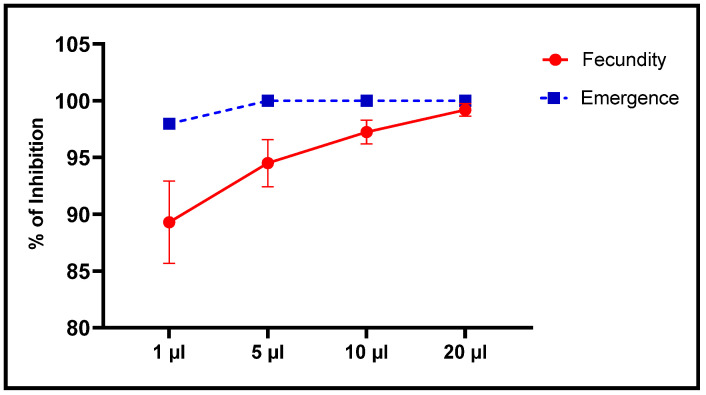
Inhibition of fecundity and emergence (means ± SD) of *C. maculatus* adults after a direct contact toxicity test with different concentrations of *L. dentata* EO.

When effects of *L. dentata* EO on oviposition by females and emergence of new *C. maculatus* individuals were also studied, the number of eggs laid was inversely proportional to concentrations of EO (Figure 9 and Figure 10). The least concentration (1 μL/L), resulted in a decrease in oviposition to 22 ± 7 eggs/female, corresponding to 89.39% lesser fecundity, compared to the control. At the greatest concentration (20 μL/L), mean number of eggs laid per female was significantly less at 1.67 ± 1.15 eggs/female, which is equivalent to 99.2% less oviposition. Mean oviposition of unexposed, female *C. maculatus* was 207.33 ± 12.5 eggs/female.

Alternatively, the number of emergences was significantly less in a dose-dependent manner with concentrations of EO to which they were exposed (Figure 9 and Figure 10). At the least concentration of 1 µL EO/L, the number of emergences of *C. maculatus* individuals after embryonic development in chickpea seeds was 3.33 ± 0.58 individuals compared to 163 ± 7.94 individuals in the control, which corresponds to 97.96% inhibition of emergence. Exposure to 5 µL EO/L resulted in 100% inhibition of emergence of new individuals.

#### 2.6.3. Repellent Activity of Essential Oil against *C. maculatus*

When the repellent activity against *C. maculatus* insects was also tested, the EO of lavender, based on the classification of McDonald (1970), exhibited only moderate repellent activity (Table 5) [29]. The rate of repulsion was dose-dependent with a maximum of 43.33 ± 5.77% observed after 120 min of exposure to the concentration of 0.315 µL/cm^2^ of the EO and a mean repulsion of 34.44% for the same period.

*L. dentata* EO exhibited significant insecticidal potency against *C. maculatus* at several levels. Mortality, oviposition and rate of emergence were all affected. These results are consistent with those of Wagner et al. (2021) [7], who observed a strong toxic potency of *L. dentata* EO against *T. castaneum* and *S. zeamais* during 6 h exposures. With the same approach, *L. angustifolia* EO exhibited significant toxic potency against two types of aphid pests of the chili and bean *M. persicae* and *A. pisum*, with a mortality of 100% after exposure to 2 µL/L in air [30].

Insecticidal effects of *L. dentata* EO against the pests studied can be attributed to its main constituents, camphor, eucalyptol and fenchone, all of which exhibited strong potency against insects of stored cereal and legume seeds, including *S. zeamais* and *T. castaneum* [31,32]. These results are consistent with those of other studies which have found that EOs, rich in camphor and eucalyptol exhibit significant toxic potency against phytophagous [33,34]. Although the mechanism of action of lavender EO on insects has was not investigated directly in this paper, studies have reported the effect of terpenoids such as eucalyptol, and camphor, which affect the nervous system, by blocking the action of the acetylcholinesterase enzyme (AChE) of insects [30,35,36,37]. In addition, a study conducted on the action of *M. arvensis* EO tested by contact against adults *S. granarius*, reported rapid paralysis and altered walking [31,38]. During the same study, essences of *M. arvensis* also induced spectacular physiological changes in treated insects, marked by up-regulation of the majority of differentially expressed proteins (DEP), which are involved in development and function of the nervous and muscular systems, protein synthesis, cellular respiration, and detoxification [39]. These findings suggest that EOs can affect a wide range of biological processes, and highlight the repair mechanisms used by surviving insects to restore the harm caused.

A significant reduction in fecundity and emergence was also observed in the insect *C. maculatus*, which was indicative of the strong ovicidal and larvicidal activity of *L. dentata* EO. Indeed, the ovicidal effect of EO tested was probably caused by blockage of embryogenesis after penetration of volatile oils into eggs through the respiratory tract of *C. maculatus* [40,41]. This is due to the direct toxicity of these compounds, which inhibit metabolic activity of eggs. This was the case for piperitone isolated from the EO of *C. schoenanthus* tested on *C. maculatus* eggs [42] and β-asarone identified in the oil of *A. calamus* tested on *C. chinensis*, *S. oryzae* and *S. granarius* eggs [43]. Further work on another bruchid species has demonstrated that the degree of sensitivity and vulnerability of eggs to the vapours of three EOs including *L. hybrida* varied according to ages of eggs and stages of embryonic development [44]. Alternatively, some reports have indicated that EOs have a sterilizing effect on eggs [43].

Results of this study demonstrated total elimination of emergence when eggs were exposed to 5 μL EO/L, which could be explained by larvicidal effects of lavender EO and their major constituent linalool. This conclusion was consistent with previous results where young larvae (L1) of the cereal seed pest *T. confusum* were most sensitive to toxic effects of *L. spica* EO and linalool, with LC_50_ = 19.535 μL/L air and LC_50_ = 14.198 μL/L air, respectively during 24 h of exposure [45]. In the same study, linalool caused greater mortality of eggs than did *L. spica* oil at equal concentrations and reduced emergence of surviving adults, larvae and pupae [45].

Repellency of *L. dentata* EO was moderate at all concentrations tested. Efficacy of EO-based repellents is usually short-lived and related to their volatility. Furthermore, synthetic repellents tend to be more effective and/or persist longer than natural repellents [46]. The degree of recursiveness of EOs is mainly due to their composition. Monoterpenes such as camphor, α-pinene, thymol and cineole are frequent components of a variety of EOs mentioned in the literature as repelling mosquitoes [47,48].

## 3. Materials and Methods

### 3.1. Chemicals

Butylated hydroxytoluene (BHT), ammonium molybdate, 2,2 diphenylpicrylhydrazyl (DPPH), quercetin, ascorbic acid, sodium phosphate, iron III chloride (FeCl_3_), potassium ferricyanide (K_3_Fe(CN)_6_) and dimethylsulphoxide (DMSO) were purchased from Sigma Aldrich (Munich, Germany). Bacterial culture media and standard antibiotics were purchased from Biokar Diagnostics (Allonne, France).

### 3.2. Plant Material

Plant material used during this study was the aerial parts, including leaves, flowers and green stems of *L. dentata*, collected around from the rural commune of Dayt Aoua (33°44′ N, 5°01′ W at 1300 m altitude) the city of Imouzzer Kandar, Morocco, at end of May 2020. Specimens were identified in the laboratory by botanists using books and plant catalogues and vouchered under the number DL78/24811. Samples were cleaned and dried in the shade and in the open air for 15 days before being extracted.

### 3.3. Extraction of Essential Oil

Using 750 mL of distilled water and a Clevenger-type apparatus, 100 g of dried aerial parts of *L. dentata* were hydro-distilled for 3 h, following procedures specified in the European Pharmacopoeia [49]. Extracted oil was dried over anhydrous sodium sulfate and kept in the dark at 4–5 °C until tested or analyzed. Based on the mass of dried plant material, the EO yield was calculated and expressed as percent (*v/w*).

### 3.4. Determination of Physical Parameters of the Essential Oil

Physical properties of EO produced from *L. dentata* were determined according to the protocol recommended by the European Pharmacopoeia [12]:

- Density: was measured using a METTLER TOLEDO 30 PX type densimeter.
- Rotatory power: was measured using an ATAGO AP300 polarimeter.
- Refractive index (n): was measured using a refractometer of the NAR-1TLIQUID type.

### 3.5. Chemical Analysis of Essential Oil

Gas chromatography coupled to a mass spectrometer (GC-MS) was used to identify the various chemical components contained in *L. dentata* EO. The investigated EO was analyzed using a Trace GC ULTRA gas-phase chromatograph with an HP-5 (5% diphenyl, 95% dimethylpolysiloxane) capillary (30 m × 00.25 mm × 00.25 µm film thickness). The detector was of the FID type set at 250 °C. The injection mode was split 1:10 with a temperature of 250 °C; Helium was utilized as the carrier gas, with a 1.4 mL/min flow rate. The column temperature was programmed with a rise of 4 °C/min from 50 to 200 °C for 5 min. The GC-MS was coupled to a Polaris Q mass spectrometer. The fragmentation was performed by electronic impact at 70 eV. Mass spectra were scanned in the range *m/z* 40–650 with an interface temperature of 300 °C. Identification of EO compounds was accomplished based on Kovats retention indices (RIs) with reference to a homologous series of *n*-alkanes and from the mass spectral database (NIST MS Library v. 2.0) [50,51].

### 3.6. In Vitro Antioxidant Activity of Essential Oil

Three different tests were performed to assess, *in vitro,* the antioxidant capacity of *L. dentate* EO: the DPPH inhibition, the reducing power (FRAP) and the total antioxidant capacity (TAC) tests.

#### 3.6.1. DPPH Free Radical Scavenging Test

The DPPH test was conducted following the adapted protocol of Moattar (2016) [52]. 750 µL of a 2,2-diphenyl-1-picrylhydrazyl (DPPH) methanolic solution (100 µM) was mixed with 100 µL of EO dissolved in methanol at various concentrations (0.1–100 mg/mL). The absorbance of the reaction mixture was measured at 517 nm after 30 min of incubation at room temperature against a negative control using methanol instead of the sample. The same test was performed with butylated hydroxytoluene (BHT) as a reference antioxidant. The percentage of DPPH radical inhibition by EO was calculated (Equation (1)).

DPPH inhibition (%) = [1 − (A/A_0_)] × 100 (1)

A and A_0_ are the absorbance of the DPPH solution in the presence and absence (negative control) of the sample respectively.

#### 3.6.2. Ferric Reducing Antioxidant Power Test (FRAP)

The FRAP test was conducted following a previously published protocol [52], in which 500 µL of phosphate buffer solution (0.2 M-pH = 6.6) and 500 µL of 1% potassium ferricyanide [K_3_Fe(CN)_6_] were added to 100 µL of varied EO concentrations (0.1–25 mg/mL) prepared in methanol. After 20 min of incubation in a water bath at 50 °C, and acidification with 500 µL of 10% trichloroacetic acid, 500 µL of distilled water and 100 µL of 0.1% FeCl_3_ were added to the reaction medium. Finally the absorbance was measured at 700 nm against a blank. The results were represented as the 50% effective concentration (EC_50_), which is the antioxidant concentration necessary to achieve an absorbance of 0.5 nm and was calculated from the graph.

#### 3.6.3. Total Antioxidant Capacity Test (TAC)

One milliliter reagent solution containing 0.6 M sulfuric acid, 28 mM sodium phosphate, and 4 mM ammonium molybdate, was combined with 25 µL EO. After incubation of the mixture for 90 min at 95 °C, the optical density was measured at 695 nm by spectrophotometer against a blank [53]. An ascorbic acid calibration curve was used to quantify the total antioxidant capacity, which was represented in milligrams of ascorbic acid equivalent per gram of EO (mg EAA/g EO). The experiment was carried out three times.

### 3.7. Antifungal Activity of Essential Oil

#### 3.7.1. Total Antioxidant Capacity Test (TAC)

Three filamentous fungi, *Alternaria alternata*, *Botrytis cinerea* and *Fusarium oxysporium* were used in this activity. All fungal strains studied are mycotoxigenic and phytopathogenic which reduce yields and quality of agricultural production. All microbial strains were provided from Hassan II Institute of Agronomy and Veterinary Sciences, Rabat, Morocco.

Spore suspensions were made from seven-day-old cultures on a potato dextrose agar (PDA) medium in a tube containing NaCl 0.9%. A Malassez cell was used to count the spores in suspension, and the suspensions were diluted to reach an inoculum concentration of approximately 10^6^ spores/mL [54].

#### 3.7.2. Disk Diffusion Method

Evaluation of the antifungal activity of *L. dentata* EO was performed by the disc diffusion method [7]. Petri plates (90 mm) containing Czapek-Dox agar medium were inoculated at the center with a 100 μL spot of the inoculum prepared (10^6^ spores/mL). Afterwards, sterile Whatman paper discs of 5 mm were deposited on the culture media surface immediately after being impregnated with EO at different concentrations (5, 10 et 40 μg/mL) prepared in dimethyl sulfoxide (DMSO). Petri plates impregnated with DMSO (10 μL per disc) were also inoculated as negative growth control. Inoculated plates were sealed with parafilm and incubated for 6 days at 25 °C [55]. For each concentration of the EO, three replicas were prepared. Daily mycelial growth was measured, and growth inhibition rates compared to the control were calculated (Equation (2)).

Mycelial growth inhibition (%) = [(dc − dt)/dc] × 100(2)

dc and dt represent the mean diameter (mm) of mycelial growth of the control and treated fungal strains, respectively.

### 3.8. Insecticidal Activity of Essential Oil

#### 3.8.1. Test Insect Collection and Rearing Condition

To test insecticidal activity of lavender EO, the chickpea pest *Callosobruchus maculatus* was studied. Rearing of the insect bruchids was conducted in glass jars containing *Cicer arietinum* chickpea seeds. The jars were kept at a constant temperature of 25 °C, relative humidity of 65 ± 5%, and a photoperiod of 14 h (light)/10 h (dark) for numerous generations.

#### 3.8.2. Toxicity of Essential Oil against *C. maculatus*

##### Toxicity by Contact

Several preliminary assays were performed to select the best doses for definitive tests. Concentrations of EO, relative to air volume in the jar used, are given in μL/L of air. In each Petri plate containing 100 g of chickpea seeds and a filter paper disc impregnated with four different concentrations of *L. dentate* EO (1, 5, 10 and 20 μL/L), 10 adult of *C. maculatus* insects (5 males and 5 females) fresh from their rearing environment and aged up to 24 h (after seed emergence) were introduced. The plates were then resealed immediately. Three replicates were performed for each concentration [56].

In order to evaluate the mortality, numbers of dead insects were counted every 24 h for four days. Eggs deposited on seeds and walls of plates were also counted using a binocular magnifying glass, followed by a regular count of emerged insects from the 28th day after confinement. The results are compared with those of the controls to calculate the rate of reduction of oviposition and emergence [56]. Mortality, corrected by use of Abbott’s formula was calculated (Equation (3)).

(3) $$Mortality\;\left(\%\right)=100\times \frac{Po-Pc}{100-Pc}$$

*Po* and *Pc* represent the observed mortality in the test and control respectively. 

The egg-laying reduction rate was calculated (Equation (4)).

(4) $$Reduction\;of\;egg\;laying\;\left(\%\right)=100\times \frac{Nc-Nt}{Nc}$$

*Nt* and *Nc* represent the number of eggs in the test and control jars respectively.

The reduction rate of emerged insects was calculated (Equation (5)).

(5) $$Reduction\;of\;emergence\;\left(\%\right)=100\times \frac{Nc-Nt}{Nc}$$

*Nc* and *Nt* represent the number of insects hatched in the control and test jars respectively.

##### Toxicity by Inhalation

Briefly, small cotton loops were suspended in glass jars, on which 1, 5, 10 or 20 μL of *L. dentata* EO were deposited by use of a micropipette. Then, ten *C. maculatus* bruchids (males and females), with age not exceeding 48 h, were placed in every jar. Then, the jars were hermetically sealed. Three replicates were performed for each experiment. The test was compared with a control (cotton without EO solution). Mortality rate was calculated as previously [56].

#### 3.8.3. Repellent Test

Repellent activity of *L. dentata* EO against *C. maculatus* insects was assessed using the preferential area method performed on filter paper described previously [29]. Filter paper discs with a diameter of 9 cm were split into two equal halves with a surface area of 31.80 cm^2^ and inserted into Petri plates. On one of the two halves a 0.5 mL volume of every concentration of EO prepared in acetone (5, 10 and 20 µL/mL of acetone) was spread uniformly, corresponding to doses of 0.079; 0.157 and 0.315 µL/cm² per disc respectively. The other half was treated only with 0.5 mL of acetone. Subsequently, 10 pairs of adult bruchids (less than 24 h old) were deposited in the center of each plate. Finally, the Petri plates were sealed with Parafilm. For each experiment, three repetitions were carried out in the same conditions as the insect rearing. The number of bruchids present on the section of the disc treated with EO was determined after 30 min and compared to the control treated with acetone.

The % of repulsion was calculated (Equation (6)) [57]:

(6) $$Repulsion\;\left(\%\right)=\frac{NC-NT}{NC+NT}\times 100$$

*NC* and *NT* represent the number of insects present in the control area and the treated area respectively.

The average repulsion percentage determined for lavender EO was allocated to one of the repulsive classes ranging from 0 to 100% [38].

### 3.9. Statistical Analysis

Mean values and standard deviations were calculated using the GraphPad Prism 8 (Microsoft Software; California, USA). The results of different tests were compared using one-way ANOVA using a Tukey-test, with the same software. The difference at *p* < 0.05 was considered to be significant. Lethal concentrations LC_50_ and LC_95_ with their confidence intervals were calculated using the Probit method [58].

## 4. Conclusions

In conclusion, the findings suggest that *L. dentata* EO has antioxidant activity, which might be beneficial for therapeutic purposes in preventing adverse effects of ROS, which explains their potency for treatment of various inflammatory illnesses. Moreover, the results demonstrated that lavender essences exhibit interesting potent antifungal and insecticidal activities, which supports their promising use as potential antifungal and bio-insecticides in agricultural production and storage of leguminous crops. The biological activities could be assigned in part to the presence of oxygenated monoterpenes.

## Figures and Tables

**Table 1:** Physical parameters of *L. dentata* EO compared to AFNOR standards.

Physical Parameters	Essential Oils	AFNOR Standards
Relative density at 20 °C	0.899	0.891 ≤ d ≤ 0.899
Refractive index at 20 °C	1.463	1.463 ≤ n ≤ 1.468
Rotatory power at 20 °C	−3.0°	−7.0° ≤ α ≤ −3.0°

**Table 2:** Phytochemical compounds of the EO extracted from *L. dentata*.

Peak	RT	Compounds	Chemical Classes	RI		Area (%)
				Cal	Lit	
1	4.98	Limonene	Monoterpene (MT)	1028	1029	0.58
2	5.01	1,8-Cineole	MT	1030	1031	7.24
3	5.41	Cis-Linalool oxide	MT	1070	1072	1.08
4	5.57	Trans-Linalooloxide	MT	1085	1086	1.06
5	5.67	Linalool	MT	1090	1090	45.06
6	6.16	Camphor	MT	1145	1146	15.62
7	6.28	Lavandulol	MT	1160	1161	1.22
8	6.33	Borneol	MT	1168	1169	8.28
9	6.42	γ-Terpineol	MT	1166	1166	7.01
10	6.47	Hexenyl butanoate	Other (O)	1185	1186	0.47
11	6.53	α-Terpineol	MT	1198	1199	1.54
12	7.01	Linalool acetate	O	1233	1234	6.01
13	7.28	Lavandulyl acetate	O	1290	1290	1.09
14	7.31	Thymol	MT	1290	1290	1.68
15	7.40	Carvacrol	MT	1298	1299	0.81
16	8.49	β-Farnesene	Sesquiterpene (ST)	1441	1442	0.86
**Chemical Classes(% by mass)**						
Monoterpene (MT)						91.18
Sesquiterpene (ST)						0.86
Others (O)						7.57
Total						99.61

RT = Retention time in minutes; RI = Retention indices; Cal = Calculate; Lit = Literature.

**Table 3:** Antioxidant activities of *L. dentata* EO (means ± SD).

	DPPH (IC_50_ mg/mL)	FRAP (EC_50_ mg/mL)	TAC (mg AAE/g EO)
**Essential oil**	12.950 ± 1.300 ^a^	11.880 ± 0.225 ^a^	81.280 ± 2.278 ^a^
**BHT**	0.134 ± 0.028 ^b^	0.362 ± 0.010 ^b^	47.540 ± 1.200 ^b^
**Quercetin**	-	0.032 ± 0.003 ^c^	28.390 ± 1.248 ^c^

Values with different letters (a, b or c) in each column are significantly different (*p* < 0.05).

**Table 4:** LC_50_ and LC_95_ (μL/L air) responsible of mortality of *C. maculatus* adults in contact and inhalation toxicity tests after 24 h treatment with *L. dentata* EO.

Bioassays	LC_50_ (μL/L Air)	LC_95_ (μL/L Air)	Chi-Square (X^2^)
Inhalation test	05.90	74.83	64.68
Contact test	04.01	16.48	62.80

**Table 5:** Results of the repellent activity of *L. dentata* EO against *C. maculatus* depending on the treatment time.

	Repellent Activity at Different Doses of Essential Oil			PR Average (%)	Class *
	0.079 (µL/cm^2^)	0.157 (µL/cm^2^)	0.315 (µL/cm^2^)		
30 min	13.33 ± 5.77	20.00 ± 10.00	26.67 ± 5.77	20.00	Moderately repellent (II)
60 min	20.00 ± 10.00	33.33 ± 5.77	40.00 ± 0.00	31.11	Moderately repellent (II)
120 min	26.67 ± 5.77	33.33 ± 5.77	43.33 ± 5.77	34.44	Moderately repellent (II)

* Class of repellent effect according to the classification of McDonald (1970).

## Data Availability

Not applicable.

## References

[1] Moumni M., Romanazzi G., Najar B., Pistelli L., Amara H.B., Mezrioui K., Karous O., Chaieb I., Allagui M.B. (2021). Antifungal activity and chemical composition of seven essential oils to control the main seedborne fungi of cucurbits. Antibiotics.

[2] Dammak I., Hamdi Z., Kammoun El Euch S., Zemni H., Mliki A., Hassouna M., Lasram S. (2019). Evaluation of antifungal and anti-ochratoxigenic activities of Salvia officinalis, *Lavandula dentata* and Laurus nobilis essential oils and a major monoterpene constituent 1,8-cineole against Aspergillus carbonarius. Ind. Crops Prod..

[3] Fisher M.C., Hawkins N.J., Sanglard D., Gurr S.J. (2018). Worldwide emergence of resistance to antifungal drugs challenges human health and food security. Science.

[4] Shang Y., Hasan K., Ahammed G.J., Li M., Yin H. (2019). Applications of Nanotechnology in Plant Growth and Crop Protection: A Review. Molecules.

[5] Allali A., Rezouki S., Louasté B., Bouchelta Y., El Kamli T., Eloutassi N., Fadli M. (2020). Study of the nutritional quality and germination capacity of Cicer arietinum infested by Callosobruchus maculatus (Fab.). Plant Cell Biotechnol. Mol. Biol..

[6] Matos L.F., Barbosa D.R.e.S., da Cruz Lima E., de Andrade Dutra K., Navarro D.M.d.A.F., Alves J.L.R., Silva G.N. (2020). Chemical composition and insecticidal effect of essential oils from Illicium verum and Eugenia caryophyllus on Callosobruchus maculatus in cowpea. Ind. Crops Prod..

[7] Wagner L.S., Sequin C.J., Foti N., Campos-Soldini M.P. (2021). Insecticidal, fungicidal, phytotoxic activity and chemical composition of *Lavandula dentata* essential oil. Biocatal. Agric. Biotechnol..

[8] Bourhia M., Laasri F.E., Aourik H., Boukhris A., Ullah R., Bari A., Ali S.S., El Mzibri M., Benbacer L., Gmouh S. (2019). Antioxidant and Antiproliferative Activities of Bioactive Compounds Contained in Rosmarinus officinalis Used in the Mediterranean Diet. Evid.-Based Complement. Altern. Med..

[9] Miladi H., Slama R.B., Mili D., Zouari S., Bakhrouf A., Ammar E. (2013). Essential oil of Thymus vulgaris L. and Rosmarinus officinalis L.: Gas chromatography-mass spectrometry analysis, cytotoxicity and antioxidant properties and antibacterial activities against foodborne pathogens. Nat. Sci..

[10] Zuzarte M., Vale-Silva L., Gonçalves M.J., Cavaleiro C., Vaz S., Canhoto J., Pinto E., Salgueiro L. (2012). Antifungal activity of phenolic-rich Lavandula multifida L. Essential oil. Eur. J. Clin. Microbiol. Infect. Dis..

[11] Bachiri L., Echchegadda G., Ibijbijen J., Nassiri L. (2016). Etude Phytochimique Et Activité Antibactérienne De Deux Espèces De Lavande Autochtones Au Maroc: «Lavandula stoechas L. et *Lavandula dentata* L.». Eur. Sci. J..

[12] EDQM (2013). European Pharmacopoeia.

[13] Imelouane B., Elbachiri A., Ankit M., Benzeid H., Khedid K. (2009). Physico-chemical compositions and antimicrobial activity of essential oil of eastern moroccan *Lavandula dentata*. Int. J. Agric. Biol..

[14] Soro k.D., Majdouli K., Khabbal Y., Zaïr T. (2014). Chemical composition and antibacterial activity of Lavandula species L. *dentata* L., L. pedunculata Mill and Lavandula abrialis essential oils from Morocco against food-borne and nosocomial pathogens. Int. J. Innov. Appl. Stud..

[15] Bettaieb Rebey I., Bourgou S., Saidani Tounsi M., Fauconnier M.-L., Ksouri R. (2017). Etude de la composition chimique et de l’activité antioxydante des différents extraits de la Lavande dentée (*Lavandula dentata*). J. New Sci..

[16] Justus B., de Almeida V.P., Gonçalves M.M., da Silva Fardin de Assunção D.P., Borsato D.M., Arana A.F.M., Maia B.H.L.N.S., de Fátima Padilha de Paula J., Budel J.M., Farago P.V. (2018). Chemical composition and biological activities of the essential oil and anatomical markers of *Lavandula dentata* L. Cultivated in Brazil. Braz. Arch. Biol. Technol..

[17] Giuliani C., Bottoni M., Ascrizzi R., Milani F., Papini A., Flamini G., Fico G. (2020). *Lavandula dentata* from Italy: Analysis of Trichomes and Volatiles. Chem. Biodivers..

[18] Cutillas A.B., Carrasco A., Martinez-Gutierrez R., Tomas V., Tudela J. (2018). Thyme essential oils from Spain: Aromatic profile ascertained by GC–MS, and their antioxidant, anti-lipoxygenase and antimicrobial activities. J. Food Drug Anal..

[19] Ramzi A. (2012). Mothana Antimicrobial and antioxidant activities and gas chromatography mass spectrometry (GC/MS) analysis of the essential oils of Ajuga bracteosa Wall. ex Benth. and *Lavandula dentata* L. growing wild in Yemen. J. Med. Plants Res..

[20] Baptista R., Madureira A.M., Jorge R., Adão R., Duarte A., Duarte N., Lopes M.M., Teixeira G. (2015). Antioxidant and antimycotic activities of two native Lavandula species from Portugal. Evid.-Based Complement. Altern. Med..

[21] Viuda-Martos M., Mohamady M.A., Fernández-López J., Abd ElRazik K.A., Omer E.A., Pérez-Alvarez J.A., Sendra E. (2011). In vitro antioxidant and antibacterial activities of essentials oils obtained from Egyptian aromatic plants. Food Control.

[22] De Martinoa L., De Feoa V., Fratiannib F., Nazzaro F. (2009). Chemistry, Antioxidant, Antibacterial and Antifungal Activities of Volatile Oils and their Components. Nat. Prod. Commun..

[23] EL Moussaoui A., Bourhia M., Jawhari F.Z., Salamatullah A.M., Ullah R., Bari A., Mahmood H.M., Sohaib M., Serhii B., Rozhenko A. (2021). Chemical Profiling, Antioxidant, and Antimicrobial Activity against Drug-Resistant Microbes of Essential Oil from *Withania frutescens* L. Appl. Sci..

[24] Astapchuk I., Yakuba G., Nasonov A. (2021). Efficacy of fungicides against pathogens of apple core rot from the genera Fusarium Link, Alternaria Nees and Botrytis (Fr.) under laboratory conditions. E3S Web Conf..

[25] Angioni A., Barra A., Coroneo V., Dessi S., Cabras P. (2006). Chemical composition, seasonal variability, and antifungal activity of Lavandula stoechas L. ssp. stoechas essential oils from stem/leaves and Flowers. J. Agric. Food Chem..

[26] Zhao Y., Yang Y.H., Ye M., Wang K.B., Fan L.M., Su F.W. (2021). Chemical composition and antifungal activity of essential oil from Origanum vulgare against Botrytis cinerea. Food Chem..

[27] Lagrouh F., Dakka N., Bakri Y. (2017). The antifungal activity of Moroccan plants and the mechanism of action of secondary metabolites from plants. J. Mycol. Med..

[28] Pradebon Brondani L., Alves da Silva Neto T., Antonio Freitag R., Guerra Lund R. (2018). Evaluation of anti-enzyme properties of Origanum vulgare essential oil against oral Candida albicans. J. Mycol. Med..

[29] McDonald L.L., Guy R.H., Speirs R.D. (1970). Preliminary Evaluation of New Candidate Materials as Toxicants, Repellents, and Attractants against Stored-Product Insects.

[30] Digilio M.C., Mancini E., Voto E., De Feo V. (2008). Insecticide activity of Mediterranean essential oils. J. Plant Interact..

[31] Abdelgaleil S.A., Mohamed M.I., Badawy M.E., El-Arami S.A. (2009). Fumigant and contact toxicities of monoterpenes to *Sitophilus oryzae* (L.) and *Tribolium castaneum* (Herbst) and their inhibitory effects on acetylcholinesterase activity. J. Chem. Ecol..

[32] Nukenine E.N., Adler C., Reichmuth C. (2010). Bioactivity of fenchone and Plectranthus glandulosus oil against Prostephanus truncatus and two strains of Sitophilus zeamais. J. Appl. Entomol..

[33] Abdelgaleil S.A. (2010). Molluscicidal and insecticidal potential of monoterpenes on the white garden snail, Theba pisana (Muller) and the cotton leafworm, Spodoptera littoralis (Boisduval). Appl. Entomol. Zool..

[34] Jiang H., Wang J., Song L., Cao X., Yao X., Tang F., Yue Y. (2016). GCxGC-TOFMS analysis of essential oils composition from leaves, twigs and seeds of cinnamomum camphora L. presl and their insecticidal and repellent activities. Molecules.

[35] López M.D., Pascual-Villalobos M.J. (2010). Mode of inhibition of acetylcholinesterase by monoterpenoids and implications for pest control. Ind. Crops Prod..

[36] Rizvi S.A.H., Ling S., Tian F., Xie F., Zeng X. (2018). Toxicity and enzyme inhibition activities of the essential oil and dominant constituents derived from Artemisia absinthium L. against adult Asian citrus psyllid Diaphorina citri Kuwayama (Hemiptera: Psyllidae). Ind. Crops Prod..

[37] Shahriari M., Zibaee A., Sahebzadeh N., Shamakhi L. (2018). Effects of α-pinene, trans-anethole, and thymol as the essential oil constituents on antioxidant system and acetylcholine esterase of Ephestia kuehniella Zeller (Lepidoptera: Pyralidae). Pestic. Biochem. Physiol..

[38] Karima K.-G., Nadia L., Ferroudja M.-B. (2016). Fumigant and repellent activity of Rutaceae and Lamiaceae essential oils against Acanthoscelides obtectus Say. Afr. J. Agric. Res..

[39] Renoz F., Demeter S., Degand H., Nicolis S.C., Lebbe O., Martin H., Deneubourg J.-L., Fauconnier M.L., Morsomme P., Hance T. (2022). The modes of action of Mentha arvensis essential oil on the granary weevil Sitophilus granarius revealed by a label-free quantitative proteomic analysis. J. Pest Sci..

[40] Wightman J.A., Southgate B.J. (1982). Egg morphology, host, and probable regions of origin of the bruchids (coleoptera: Bruchidae) that infest stored pulses—An identification aid. N. Z. J. Exp. Agric..

[41] Nyamador S.W., Ketoh G.K., Koumaglo H.K., Glitho I.A. (2010). Activités Ovicide et Larvicide des Huiles Essentielles de Cymbopogon giganteus Chiov. et de Cymbopogon nardus L. Rendle sur les stades immatures de Callosobruchus maculatus F. et de Callosobruschus subinnotatus Pic. (Coleoptera: Bruchidae). J. Soc. Ouest-Afr. Chim..

[42] Ketoh G.K., Koumaglo H.K., Glitho I.A., Huignard J. (2006). Comparative effects of Cymbopogon schoenanthus essential oil and piperitone on Callosobruchus maculatus development. Fitoterapia.

[43] Schmidt G.H., Risha E.M., El-Nahal A.K.M. (1991). Reduction of progeny of some stored-product Coleoptera by vapours of Acorus calamus oil. J. Stored Prod. Res..

[44] Papachristos D.P., Stamopoulos D.C. (2004). Fumigant toxicity of three essential oils on the eggs of Acanthoscelides obtectus (Say) (Coleoptera: Bruchidae). J. Stored Prod. Res..

[45] Kheloul L., Anton S., Gadenne C., Kellouche A. (2020). Fumigant toxicity of Lavandula spica essential oil and linalool on different life stages of Tribolium confusum (Coleoptera: Tenebrionidae). J. Asia. Pac. Entomol..

[46] Fradin M.S., Day J.F. (2002). Comparative Efficacy of Insect Repellents against Mosquito Bites. N. Engl. J. Med.

[47] Jaenson T.G.T., Pålsson K., Borg-Karlson A.K. (2006). Evaluation of extracts and oils of mosquito (Diptera: Culicidae) repellent plants from Sweden and Guinea-Bissau. J. Med. Entomol..

[48] Yang P., Ma Y. (2005). Repellent effect of plant essential oils against Aedes albopictus. J. Vector Ecol..

[49] EDQM (2004). European Pharmacopoeia.

[50] El Atki Y., Aouam I., El Kamari F., Taroq A., Lyoussi B., Oumokhtar B., Abdellaoui A. (2020). Phytochemistry, antioxidant and antibacterial activities of two Moroccan Teucrium polium L. subspecies: Preventive approach against nosocomial infections. Arab. J. Chem..

[51] Adams R.P. (2007). Identification of Essential Oil Components by Gas Chromatograpy/Mass Spectrometry.

[52] Moattar F.S., Sariri R., Yaghmaee P., Giahi M. (2016). Enzymatic and non-enzymatic antioxidants of Calamintha officinalis moench extracts. J. Appl. Biotechnol. Rep..

[53] Maškovič P.Z., Manojlovič N.T., Mandič A.I., Mišan A.Ç., Milovanovic I.L., Radojkovič M.M., Cvijovič M.S., Solujič S.R. (2012). Phytochemical screening and biological activity of extracts of plant species Halacsya sendtneri (Boiss.) Dörfl. Hem. Ind..

[54] Moussa H., Hriouech S., Tanghort M., Chefchaou H., Mzabi A., Chami N., Remmal A. (2020). A comparative study of the antifungal activity of a natural product based on essential oils with imazalil and thiabendazole on Penicillium digitatum and Penicillium italicum. Plant Cell Biotechnol. Mol. Biol..

[55] El Moussaoui A., Jawhari F.Z., Almehdi A.M., Elmsellem H., Fikri Benbrahim K., Bousta D., Bari A. (2019). Antibacterial, antifungal and antioxidant activity of total polyphenols of *Withania frutescens* L. Bioorg. Chem..

[56] Aimad A., Sanae R., Anas F., Abdelfattah E.M., Bourhia M., Mohammad Salamatullah A., Alzahrani A., Alyahya H.K., Albadr N.A., Abdelkrim A. (2021). Chemical Characterization and Antioxidant, Antimicrobial, and Insecticidal Properties of Essential Oil from Mentha pulegium L. Evid.-Based Complement. Altern. Med..

[57] Zandi-Sohani N., Hojjati M., Carbonell-Barrachina Á.A. (2013). Insecticidal and Repellent Activities of the Essential Oil of Callistemon citrinus (Myrtaceae) Against Callosobruchus maculatus (F.) (Coleoptera: Bruchidae). Neotrop. Entomol..

[58] Finney D.J. (1971). Probit Analysis.

